# Accuracy of attenuation coefficient measurement (ACM) for hepatic steatosis: comparison with MRI proton density fat fraction (MRI-PDFF) and chemical fat analysis using multimodal liver fat phantoms

**DOI:** 10.3389/fmed.2025.1698952

**Published:** 2025-12-03

**Authors:** Maksym Zhaivoronok, Oleh Dynnyk, Nataliya Deresh, Tamara Nosenko, Nazarii Kobyliak

**Affiliations:** 1 Department of Nuclear Medicine, Radiation Oncology and Radiation Safety of Shupyk National Healthcare University of Ukraine, Kyiv, Ukraine; 2 “Institute of Elastography” Medical Center LLC, Kyiv, Ukraine; 3 Department of Radiology, Lifescan Diagnostic Center, Kyiv, Ukraine; 4 Department of Fat Technology, Chemical Technology of Food Additives and Cosmetics of the National University of Food Technologies, Kyiv, Ukraine; 5 Endocrinology Department, Bogomolets National Medical University, Kyiv, Ukraine

**Keywords:** ultrasound, phantom, steatosis, MRI-PDFF, attenuation coefficient measurement (ACM)

## Abstract

**Background:**

The objective of this study was to evaluate the accuracy and reproducibility of real-time ultrasound steatometry using attenuation coefficient measurement (ACM) in comparison with magnetic resonance imaging proton density fat fraction (MRI-PDFF) and chemical fat analysis. Specifically, we assessed the diagnostic performance and reproducibility of ultrasound-based ACM against MRI-PDFF and laboratory fat quantification using multimodal liver fat phantoms (LFPs) with varying fat-to-water ratios.

**Methods:**

Sixty LFPs with different fat concentrations were examined in two radiology centers and one chemical laboratory. Each phantom underwent three assessments: MRI-PDFF, ultrasound ACM, and laboratory-based chemical fat quantification. Correlation coefficients, intraclass correlation coefficients (ICC), receiver operating characteristic (ROC) analyses, and Bland–Altman analysis were performed to evaluate the relationships and agreement among the measurement methods.

**Results:**

Median values of ACM, MRI-PDFF, and laboratory fat content were 2.39 (1.87–2.84), 3.88 (2.46–7.60), and 1.24 (0.51–3.90), respectively. Strong correlations were observed between ACM and laboratory fat quantification (*r* = 0.878, *p* < 0.001) and between MRI-PDFF and laboratory analysis (*r* = 0.881, *p* < 0.001). ACM also correlated strongly with MRI-PDFF (*r* = 0.846, *p* < 0.001). The intraobserver reproducibility of ACM was excellent (ICC = 0.956, *p* < 0.001). AUROC values were 0.984 for ACM and 0.996 for MRI-PDFF, both indicating high diagnostic sensitivity and specificity.

**Conclusions:**

ACM demonstrated strong agreement with MRI-PDFF and chemical fat analysis in LFPs, supporting its potential as a reliable, accurate, and cost-effective non-invasive technique for hepatic steatosis quantification.

## Introduction

Metabolic dysfunction-associated steatotic liver disease (MASLD) represents a major and continuously rising global health concern (1). The growing incidence of MASLD underscores the urgent need for effective diagnostic techniques enabling early detection and timely intervention (2). MASLD typically results from excessive lipid accumulation within hepatocytes, which may progress to inflammation, fibrosis, and ultimately cirrhosis. Its rising prevalence—closely linked to modern lifestyle and dietary patterns—highlights the importance of developing accurate and efficient diagnostic methods for the prompt identification of MASLD (3).

In its early stages, MASLD is often asymptomatic, which complicates its detection and diagnosis. A histopathological hallmark of steatotic liver disease (SLD) is observed when the proportion of hepatocytes containing fat vacuoles exceeds 5% of the total hepatocyte population in the liver (4). However, if SLD remains undetected and preventive or therapeutic interventions are not implemented at an early stage, the condition may progress to metabolic dysfunction-associated steatohepatitis (MASH), eventually leading to fibrosis, cirrhosis, and hepatocellular carcinoma (5). Radiologic imaging modalities, including ultrasound (US), computed tomography (CT), and magnetic resonance imaging —particularly MRI-PDFF assessment—serve as essential non-invasive tools for the detection and staging of SLD (6). Furthermore, these imaging techniques provide comprehensive insights into hepatic morphology and facilitate the identification of localized fat deposition patterns, such as focal steatosis and fat-sparing areas (7, 8). Recent clinical studies have confirmed that ultrasound-based attenuation imaging correlates strongly with MRI-PDFF, supporting its use as a quantitative biomarker of hepatic steatosis (9).

MRI demonstrates excellent reproducibility across different platforms and manufacturers in liver fat quantification using MRI-PDFF technology (10). Compared with liver biopsy, various MRI-based techniques have demonstrated reliable quantification of hepatic steatosis in patients with MASLD (11). Importantly, the sensitivity of MRI-PDFF to detect mild hepatic steatosis is crucial for the early diagnosis of SLD (12).

MRI-PDFF has emerged as a reliable non-invasive standard for hepatic fat quantification, offering high reproducibility and cross-platform consistency. However, despite its accuracy, few studies have directly compared imaging-based steatometry with physical or chemical fat quantification in controlled liver-mimicking models. Incorporating laboratory-based fat analysis as an independent reference standard provides a more objective validation framework for assessing the diagnostic accuracy of US attenuation measurements.

However, despite significant progress in quantitative ultrasound research, previous studies have mainly relied on MRI-based validation and lacked standardized physical models that allow reproducible calibration and cross-technology comparison. Incorporating laboratory-based chemical fat quantification provides an objective validation framework that bridges this methodological gap and supports accurate benchmarking of ultrasound steatometry techniques.

The aim of this study was to evaluate the accuracy and reproducibility of ultrasound ACM in comparison with MRI-PDFF steatometry and laboratory-based chemical fat quantification in a series of LFPs with varying fat-to-water ratios.

## Materials and methods

### Study design

A comparative evaluation of SLD imaging methods was performed using a LFP simulating various fat-to-water ratios, which was developed at a medical center. Two radiology centers and a fat analysis laboratory participated in the study. Another medical center conducted MRI examinations of the same LFP using a 1.5 T scanner. The fat content of samples from the same LFP was determined in the laboratory by exhaustive fat extraction using petroleum ether as the solvent. Ultrasound ACM was performed five times for each LFP volume, and median values were calculated. On the same day, MRI-PDFF assessments and laboratory fat composition analyses were conducted using the same LFP samples.

**Figure 1:**
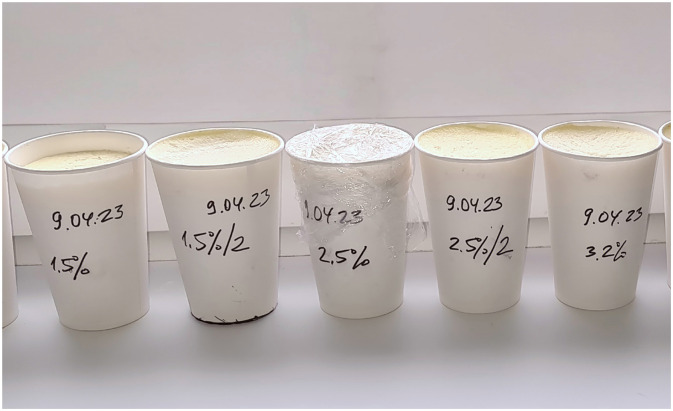
Paper cups filled with semolina-based mixtures of milk or cream (1%−20% fat) used as LFP to simulate different degrees of steatosis. Labels indicate preparation date and fat content.

A multimodal phantom structure was developed using a container made of non-magnetic material (plastic or waxed paper). A 300-mL beaker was filled with a mixture of semolina, water, and a fat suspension (either natural milk or cream) in varying proportions. The beaker had an open top to allow acoustic coupling with the ultrasound transducer. A flat sponge (foam rubber) 3–4 mm thick was soaked in water and placed at the bottom of the beaker to serve as a damper, reducing ultrasonic reverberation artifacts. Boiled semolina produced a finely granulated homogeneous pattern in B-mode, resembling the echotexture of liver parenchyma. For this study, 60 LFPs were prepared with different concentrations of whole milk diluted with water (1%, 2.5%, 3.2%) and cream ranging from 5% to 20%. The natural composition of milk consists of a suspension of spherical triglyceride droplets stabilized by proteins, closely resembling hepatocellular fat vacuoles (0.2–10 μm) (13). The ultrasound-based steatometry method using ACM relies on evaluating the integral attenuation of the US signal within the medium, which results from reflection, scattering, and absorption of ultrasonic waves of specific frequencies by fat droplets of varying sizes and concentrations. By adjusting the concentration of milk with different fat contents, various degrees of hepatic steatosis were simulated (Figure 1).

Researchers performing diagnostic and laboratory assessments were not informed of the exact milk concentrations in advance, ensuring that the study remained blinded. The inherent variability and uncertainty of fat content within the liver fat phantoms (LFPs) closely simulated real-world conditions, reflecting the unpredictability of hepatic steatosis severity in clinical settings, where the examiner cannot anticipate the degree of liver steatosis beforehand. To minimize temporal variability, all imaging and chemical analyses were performed within 48 h after phantom preparation. Due to the use of natural ingredients, the shelf life of each LFP was limited to 2–3 days, after which the phantoms were no longer suitable for use.

### US steatometry

We conducted an investigation using our LFP in accordance with the standardized human US steatometry protocol. The US transducer was positioned perpendicularly to the upper surface of the LFP, with the phantom's surface displayed horizontally on the device monitor. An acoustic window was used to obtain optimal B-mode imaging quality.

The image capture was performed only in areas where the selected ROI was free from artifacts such as reverberations and shadowing, enabling accurate ACM. Modern US systems facilitate precise ROI navigation and artifact avoidance to enhance measurement reliability and reproducibility. To assess the ACM, we utilized specific parameters and US scanning settings tailored for evaluating LS with the Soneus P7 stationary US system. The region of interest (ROI) was positioned at a standardized depth of 5–6 cm, measured from the phantom surface to the center of the ROI, in accordance with WFUMB and EFSUMB quantitative ultrasound recommendations (14). This depth range was selected to ensure optimal acoustic penetration, minimize near-field and reverberation artifacts, and maintain a stable signal-to-noise ratio for ACM. The lateral width of the ROI was fixed at 4.5 cm. Standardized ROI navigation for ACM was guided by a specialized attenuation profilogram displayed on the system interface, enabling consistent positioning across all measurements. The upper boundary of the ROI for the ACM was set 1 cm beneath the LFP surface to minimize interference from reverberation artifacts originating from the anterior plane of the LFP. The median ACM value was calculated from five consecutive measurements (15).

**Figure 2:**
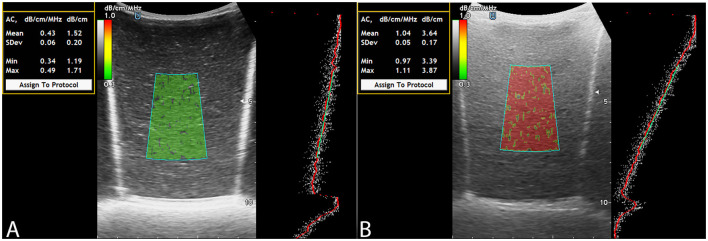
ACM: fat-free LFP **(A)**, LFP filled with fat solution **(B)**.

To evaluate the visual and quantitative expression of fat content using ACM, color mapping patterns in the LFPs were analyzed. The color of the ROI in ACM mode on the Soneus 7 system changed progressively with increasing fat concentration, closely resembling the appearance of hepatic parenchyma in clinical imaging. Phantoms with minimal fat exhibited low attenuation and were characterized by a uniform green hue, whereas higher fat concentrations resulted in progressively greater attenuation and a shift in the color map toward yellow and red shades (Figure 2).

### MRI steatometry

**Figure 3:**
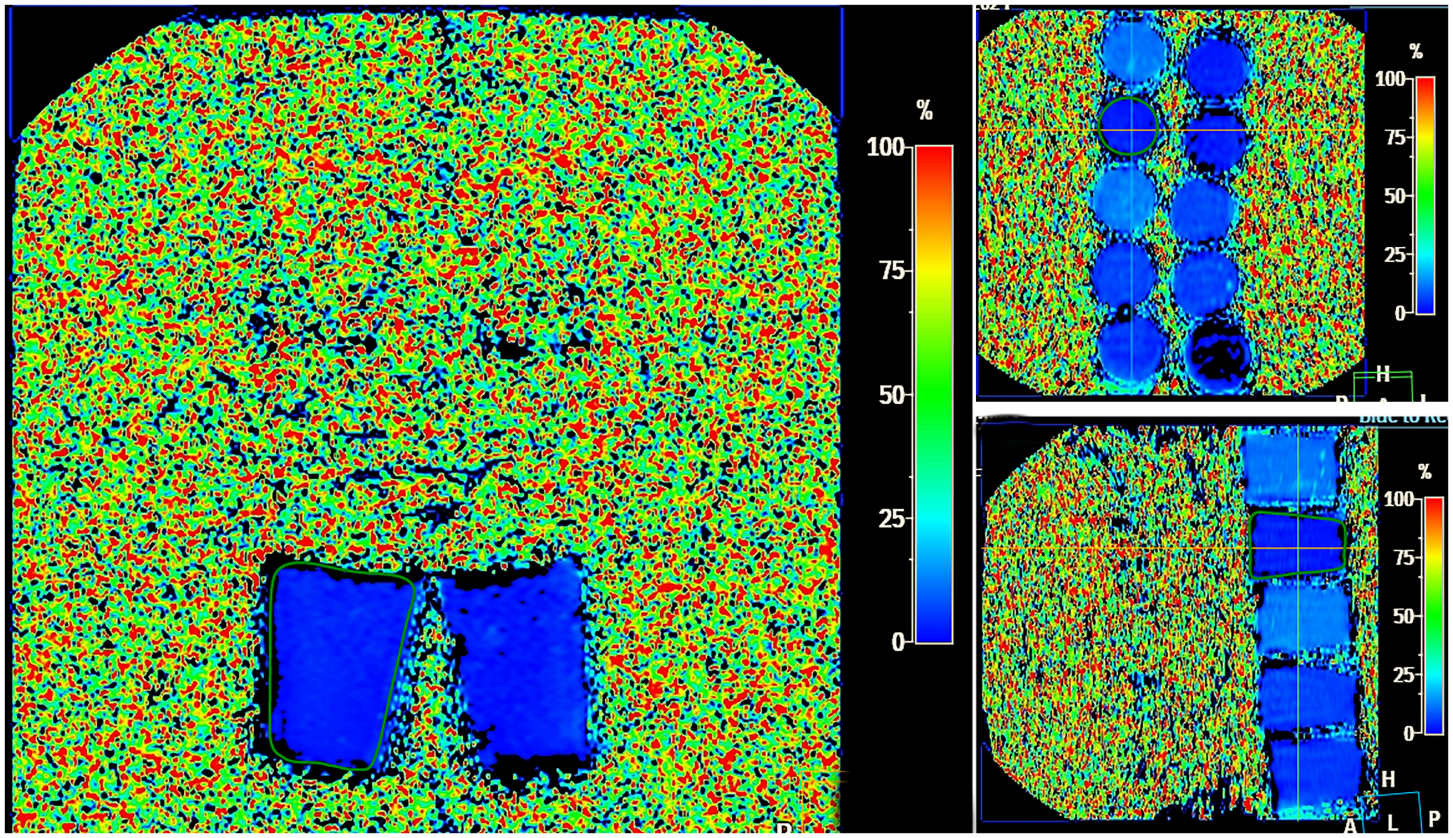
MRI steatometry mDIXON_Quant, Liver Health postprocessing—color map showing different degrees of fat in LFP in the FF range from 0%−100% (shades from dark blue–light blue).

MRI was performed using a Philips Ingenia 1.5 T magnetic resonance system equipped with a multichannel abdominal coil. The protocol for LFP quantification included five imaging series: water-only T1-TFE (W), fat-only T1-TFE (F), transverse relaxation rate maps (R2 and T2^*^),^*^ and fat fraction (FF) mapping. Acquisition parameters were as follows: flip angle, 5°; field of view (FOV), 400 mm; slice thickness, 6.0 mm; slice gap, −3.0 mm. Post-processing was performed on a dedicated workstation and included segmental liver volumetry (cm3), calculation of mean fat fraction (FF %), transverse relaxation times (T2, R2), and generation of histograms for quantitative analysis (Figure 3).

### Gravimetric determination of fat content

Approximately 15 g of the model sample was weighed in a metal container and dried at 105 °C until it reached a stable mass. The dry samples were quantitatively transferred to filter paper shells, weighed and placed in a Soxhlet extractor. The fat was extracted with n-hexane in a water bath for 20 h. The duration of the extraction cycle was approximately 20 min. The shells were withdrawn from the extractor, dried and weighed after extraction. The fat content was calculated according to the following equation:


$$\begin{array}{l} \text{F}=\frac{\left({\text{m}}_{\text{i}}-{\text{m}}_{\text{f}}\right)}{\text{m}}.100, \end{array}$$

where *m*_*i*_ represents the initial mass of the paper shell, g; *m*_*f*_ represents the mass of the paper shell after extraction, g; and *m* represents the mass of the sample, g.

### Statistical analysis

Quantitative data were expressed as the median and interquartile range (25th−75th percentiles). The normality of variable distributions was assessed using the Shapiro–Wilk test. A *p-value* > 0.05 indicated a distribution consistent with normality, whereas *p* ≤ 0.05 suggested deviation from normality. The Pearson correlation coefficient (CC) (r) was calculated to evaluate the strength of association between variables. Correlation strength was classified as strong for *r* > 0.8 and excellent for *r* > 0.9. A *p-value* < 0.05 was considered statistically significant. The intraclass correlation coefficient (ICC) was computed to assess intraobserver reproducibility, since all ACM measurements were performed by a single experienced operator. ICC values were interpreted as follows: < 0.5 — poor, 0.5–0.75 — moderate, 0.75–0.9 — good, and >0.9 — excellent consistency. A *p-value* < 0.05 was considered statistically significant. To further evaluate agreement between quantitative methods (MRI-PDFF, ACM, and chemical fat analysis), a Bland–Altman analysis was performed. Mean bias and 95% limits of agreement were calculated and presented graphically to visualize systematic differences and variability between measurement techniques.

The laboratory determination of fat content served as the reference standard for calculating the area under the receiver operating characteristic curve (AUROC). ROC analysis was performed to evaluate the diagnostic performance of the ACM. For ROC analysis, the continuous ACM and MRI-PDFF values were binarized according to the histological definition of steatosis (≤5% — normal, >5% — steatotic), providing the reference framework for assessing diagnostic accuracy. The AUROC value was calculated to assess the overall accuracy of the diagnostic model, while sensitivity and specificity were derived from optimal threshold values determined by the Youden index.

## Results

**Figure 4:**
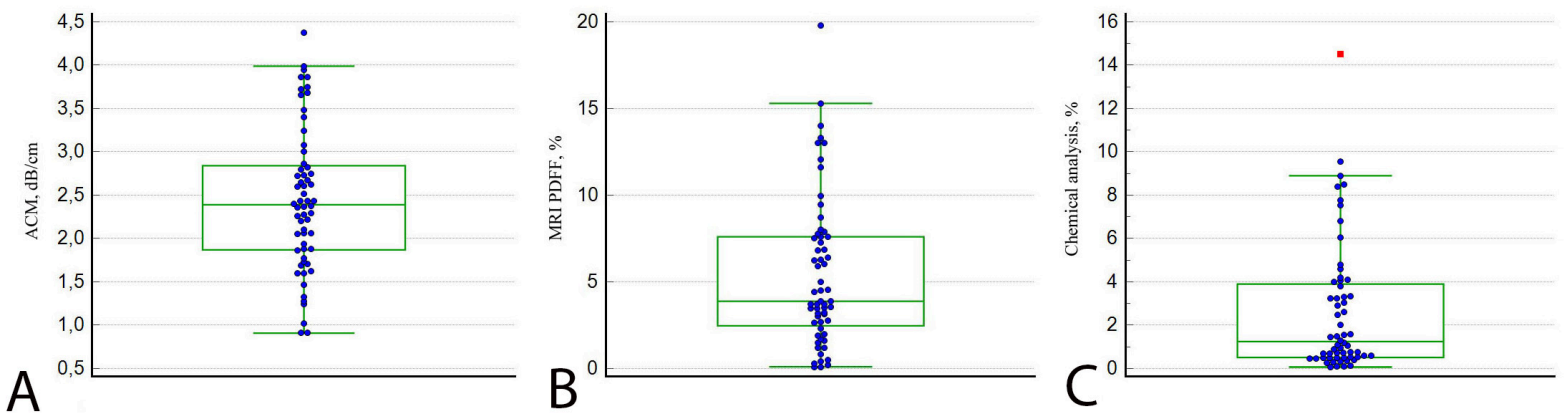
Plots of LFP measurements by ACM **(A)**, MRI-PDFF **(B)**, and chemical analysis **(C)**. The lines in each diagram represent the median value.

The B-mode appearance of the LFP closely resembled that of normal liver parenchyma, displaying a homogeneous, fine-grained gray echotexture. The overall median values (25th−75th percentiles) of the ACM (dB/cm), fat fraction determined by MRI-PDFF (%), and chemically quantified fat content in the LFPs (%) were 2.39 (1.87–2.84), 3.88 (2.46–7.60), and 1.24 (0.51–3.90), respectively (Figure 4).

**Figure 5:**
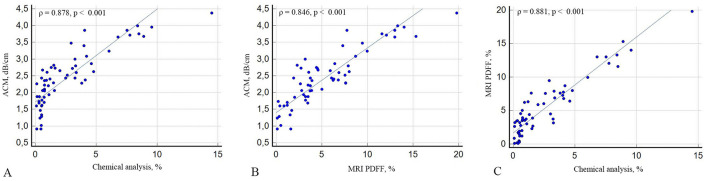
Correlation analysis between ACM by chemical analysis **(A)**, ACM vs. MRI PDFF **(B)** and MRI-PDFF vs. chemical analysis **(C)**.

The laboratory fat content, used as the reference standard, correlated strongly with ACM (ρ = 0.878; 95% CI: 0.803–0.925; *p* < 0.001) and MRI-PDFF (ρ = 0.881; 95% CI: 0.805–0.926; *p* < 0.001), respectively. Likewise, ACM and MRI-PDFF demonstrated a strong positive correlation (ρ = 0.846; 95% CI: 0.754–0.909; *p* < 0.001; Figure 5). The ICC for ACM demonstrated excellent intraobserver reproducibility, with a value of 0.956 (95% CI: 0.917–0.981; *p* < 0.001). Comparative analysis using the Mann–Whitney U-test revealed a statistically significant difference between normal and steatotic liver fat phantoms for both ACM (*p* < 0.001) and MRI-PDFF (p < 0.001). These findings confirm that both imaging modalities effectively distinguish between different fat content levels.

**Figure 6:**
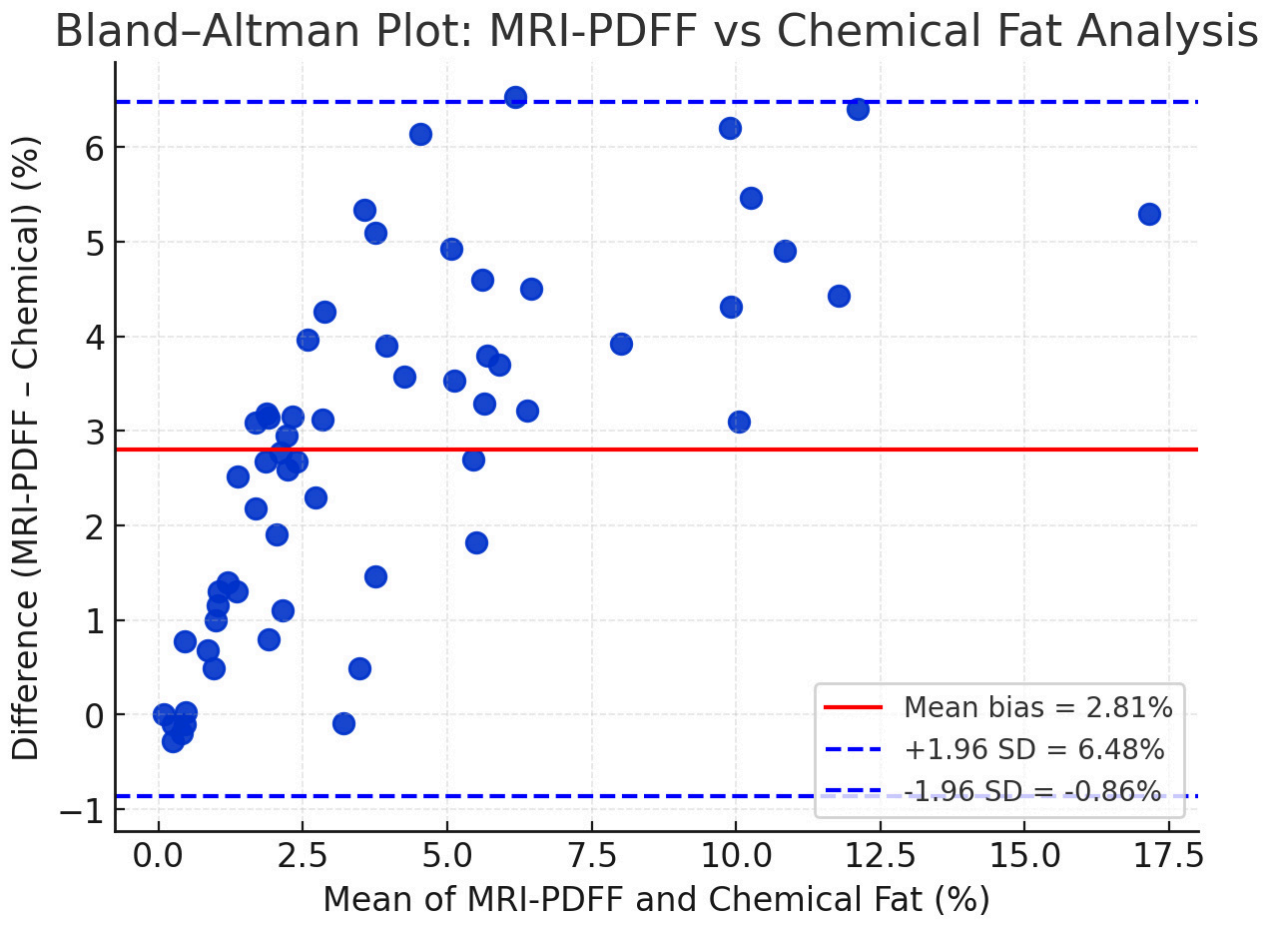
Bland–Altman plot comparing MRI-PDFF and laboratory fat analysis.

Bland–Altman analysis was performed to evaluate the agreement between MRI-PDFF and laboratory-based chemical fat quantification, as both methods express hepatic fat content in percentage (%). The analysis demonstrated a mean bias of +2.81%, with 95% limits of agreement ranging from −0.86% to +6.48% (Figure 6). These findings indicate that MRI-PDFF slightly overestimated fat content relative to the chemical reference method. However, the differences remained within narrow and clinically acceptable limits, confirming excellent concordance between MRI-based and laboratory quantification of hepatic fat. The graphical distribution of the data points did not reveal any proportional bias, suggesting homogeneity of measurement differences across the full range of fat content values.

**Figure 7:**
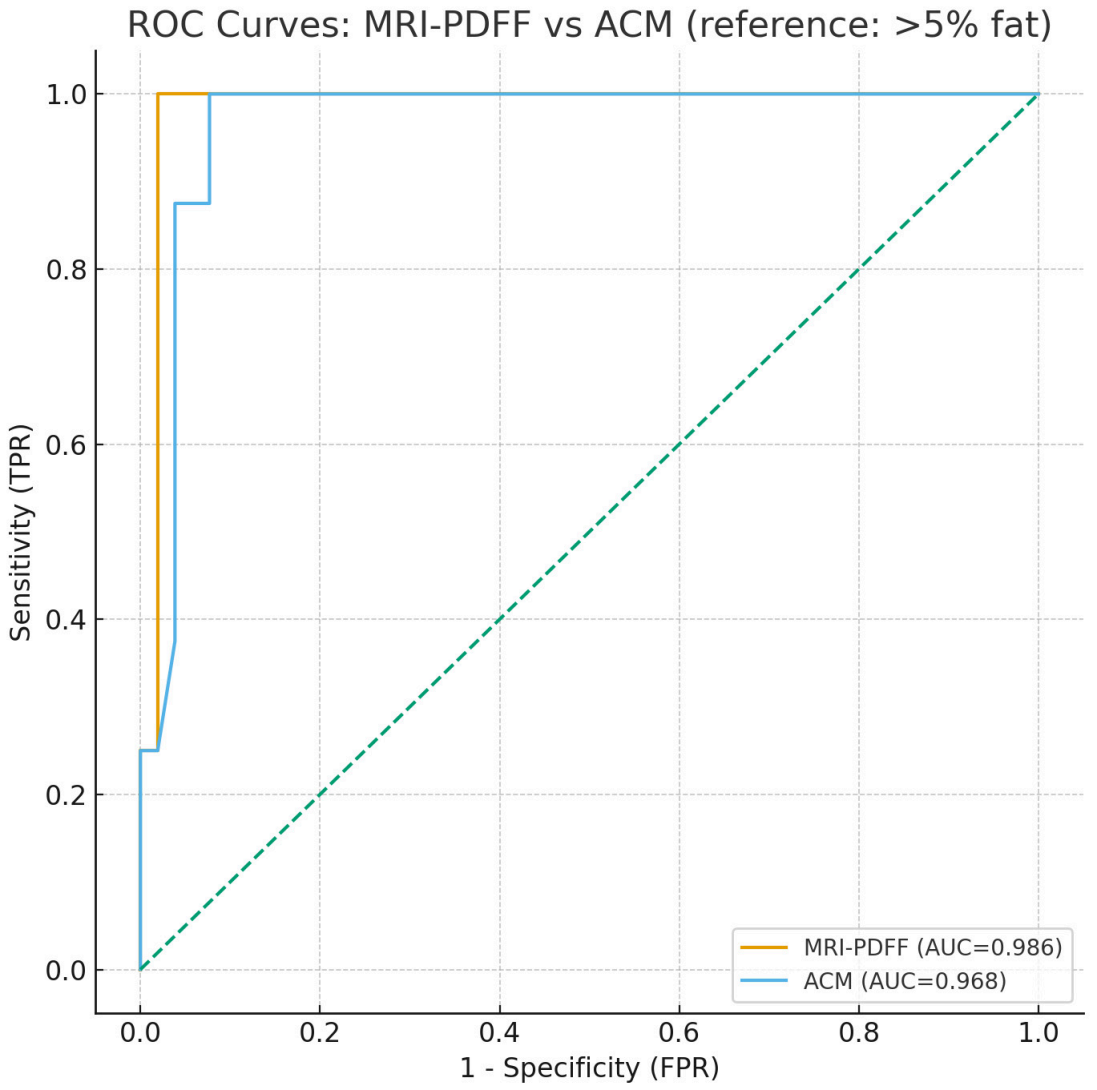
Receiver operating characteristic (ROC) curves illustrating the diagnostic performance of ACM and MRI-PDFF for detecting hepatic steatosis, using laboratory fat content as the reference standard.

The AUROC for ACM was 0.968 (95% CI: 0.916–1.000; *p* < 0.001), with a sensitivity of 100% (95% CI: 66.4–100.0; *p* < 0.001) and a specificity of 94.1% (95% CI: 83.8–98.8; *p* < 0.001). For MRI-PDFF, the AUROC was 0.986 (95% CI: 0.950–1.000; *p* < 0.001), with a sensitivity of 100% (95% CI: 63.1–100.0; *p* < 0.001), and a specificity of 98.0% (95% CI: 89.7–100.0; *p* < 0.001; Figure 7).

## Discussion

Previous research by Kobyliak et al. (10) utilized LFPs to evaluate ultrasound ACM, computed tomography (CT), and MRI-PDFF for the non-invasive quantification of hepatic steatosis. Building upon this methodology, our study further incorporated laboratory-based chemical fat analysis, thereby providing an additional and objective reference standard for validation of imaging-based fat quantification.

Radiological imaging techniques—including US, CT, MRI, and elastography—are increasingly recognized as reliable and widely adopted non-invasive alternatives to liver biopsy for the diagnosis and management of SLD (16–18). These imaging modalities enable accurate and quantitative assessment of key pathological features, including hepatic steatosis and progressive liver fibrosis (19). In line with previous work, Di Lascio et al. (20) proposed the Steato-Score, a composite ultrasound index for quantifying liver fat content, which demonstrated a strong correlation with MRI-PDFF and provided an accurate, noninvasive alternative for assessing hepatic steatosis. These findings further support the clinical relevance of quantitative ultrasound approaches in evaluating liver fat burden. A major advancement in abdominal US is the ACM, which enables quantitative assessment and staging of SLD (15). One of the main points of discussion is which radiological technology—US, CT, or MRI—is best suited for different clinical applications, such as screening, primary and secondary diagnosis, liver transplant evaluation, or monitoring disease progression, and the effectiveness of SLD treatment (21). Currently, the evaluation of US capabilities in assessing SLD—both semiquantitative (traditional B-mode imaging, the Hamaguchi scoring system, and the hepatorenal index) and quantitative (ACM, backscatter analysis, and ultrasound wave velocity within the liver parenchyma)—is primarily based on comparative studies using MRI-PDFF as the reference standard (22).

The criteria for high-quality visualization and accurate ROI navigation include color coding, attenuation degree, and attenuation depth graphs (profilograms). These technologies facilitate clear delineation of the liver parenchyma, enabling the operator to avoid artifacts such as reverberations and acoustic shadows, and to exclude measurements from unwanted anatomical structures. Such technical features of modern US systems may enhance measurement performance and overall diagnostic accuracy (10). Reducing the cost of US technology—particularly through the development of portable and handheld devices equipped with ACM capabilities—facilitates broader implementation in population-based studies of SLD and supports point-of-care diagnostic protocols. In a recent pilot study, handheld point-of-care ultrasound systems (POCUS) were successfully utilized to measure the attenuation and backscatter coefficients, demonstrating both feasibility and validity when compared with MRI-PDFF in adults at risk for SLD (23).

Our study, in line with previous research, demonstrates that ACM is an informative and accessible technique for the early detection and staging of SLD. Accurate and non-invasive stratification of hepatic steatosis therefore represents a topic of substantial clinical importance. The key advantage of US systems lies in their cost-effectiveness and widespread availability compared with other radiological modalities. These results further confirm the discriminative reliability of ACM in differentiating normal and steatotic fat content levels, supporting its diagnostic value for quantitative assessment of hepatic steatosis.

LFPs facilitate the training process for emerging US technologies, enabling operators, including beginners, to acquire practical skills more efficiently. The absence of typical scanning challenges—such as artifacts from ribs or gas within the lungs and intestines—allows the operator to concentrate on the essential aspects of steatometry, thereby improving measurement accuracy and reproducibility.

This study has several limitations. First, it was conducted using LFPs that, although closely simulating hepatic parenchyma, cannot fully reproduce *in vivo* physiological conditions such as perfusion and respiratory motion. Second, the natural composition of the LFPs limited their stability to 2–3 days, which may have influenced measurement reproducibility. Third, all ACM assessments were performed by a single operator, precluding evaluation of interobserver variability. Additionally, the study utilized a single US system (Soneus P7), and further cross-platform validation is warranted. Finally, the absence of *in vivo* data limits the direct clinical translation of the proposed findings, which should be addressed in future patient-based studies.

Future studies should focus on clinical validation of ACM in real-world patient populations with varying degrees of hepatic steatosis. Such investigations will help confirm the diagnostic performance of ACM under physiological conditions and facilitate its broader implementation as a quantitative ultrasound tool for non-invasive liver fat assessment.

## Conclusions

Modeling liver steatosis using multimodal LFPs with varying fat-to-water ratios demonstrated the high accuracy and reproducibility of ACM compared with MRI-PDFF and laboratory-based chemical fat analysis. ACM showed a strong correlation with both MRI-PDFF and laboratory results, confirming its potential as a robust, accurate, and cost-effective ultrasound-based biomarker for quantifying hepatic steatosis. Furthermore, the use of LFPs contributes to the methodological standardization and training in quantitative ultrasound steatometry.

## Data Availability

The original contributions presented in the study are included in the article/supplementary material, further inquiries can be directed to the corresponding author.
